# Blood glucose concentration is unchanged during exposure to acute normobaric hypoxia in healthy humans

**DOI:** 10.14814/phy2.14932

**Published:** 2021-08-02

**Authors:** Jason S. Chan, Alexandra E. Chiew, Alexander N. Rimke, Garrick Chan, Zahrah H. Rampuri, Mackenzie D. Kozak, Normand G. Boulé, Craig D. Steinback, Margie H. Davenport, Trevor A. Day

**Affiliations:** ^1^ Department of Biology Faculty of Science and Technology Mount Royal University Calgary AB Canada; ^2^ Alberta Diabetes Institute Faculty of Kinesiology, Sport, and Recreation University of Alberta Edmonton AB Canada

**Keywords:** acute hyperglycemia, acute hypoxia, blood [glucose] regulation, insulin sensitivity

## Abstract

Normal blood [glucose] regulation is critical to support metabolism, particularly in contexts of metabolic stressors (e.g., exercise, high altitude hypoxia). Data regarding blood [glucose] regulation in hypoxia are inconclusive. We aimed to characterize blood [glucose] over 80 min following glucose ingestion during both normoxia and acute normobaric hypoxia. In a randomized cross‐over design, on two separate days, 28 healthy participants (16 females; 21.8 ± 1.6 years; BMI 22.8 ± 2.5 kg/m^2^) were randomly exposed to either NX (room air; fraction of inspired [F_I_]O_2_ ~0.21) or HX (F_I_O_2_ ~0.148) in a normobaric hypoxia chamber. Measured F_I_O_2_ and peripheral oxygen saturation were both lower at baseline in hypoxia (*p *< 0.001), which was maintained over 80 min, confirming the hypoxic intervention. Following a 10‐min baseline (BL) under both conditions, participants consumed a standardized glucose beverage (75 g, 296 ml) and blood [glucose] and physiological variables were measured at BL intermittently over 80 min. Blood [glucose] was measured from finger capillary samples via glucometer. Initial fasted blood [glucose] was not different between trials (NX:4.8 ± 0.4 vs. HX:4.9 ± 0.4 mmol/L; *p* = 0.47). Blood [glucose] was sampled every 10 min (absolute, delta, and percent change) following glucose ingestion over 80 min, and was not different between conditions (*p* > 0.77). In addition, mean, peak, and time‐to‐peak responses during the 80 min were not different between conditions (*p* > 0.14). There were also no sex differences in these blood [glucose] responses in hypoxia. We conclude that glucose regulation is unchanged in young, healthy participants with exposure to acute steady‐state normobaric hypoxia, likely due to counterbalancing mechanisms underlying blood [glucose] regulation in hypoxia.

## Graphical abstract

In a large sample of young healthy male and female participants, blood glucose homeostasis in response to a standard oral glucose load (75 g, 296 ml) was unchanged in response to exposure to acute normobaric hypoxia (FIO2 14.8%) compared to room air over 80‐min, using multiple metrics including mean, peak, and time‐to‐peak responses.

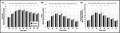

## 1 INTRODUCTION

Blood glucose concentration is tightly regulated through well‐established mechanisms, which are essential for maintaining metabolic homeostasis. Many factors, including carbohydrate ingestion, exercise, stress, and environmental conditions may affect glucose regulation in humans (Ahlborg et al., 1974; Kamba et al., 2016; Larsen et al., 1997). Responses to these factors are important for the maintenance of metabolic homeostasis, and serious consequences may result following significant acute or chronic changes in glucose regulation, such as metabolic diseases.

The ingestion of carbohydrates and subsequent absorption into the bloodstream results in a temporary rise in blood glucose. Normally, this rise in blood glucose is associated with a concomitant rise in insulin, released from pancreatic beta cells, lowering blood glucose again via uptake, utilization, and/or storage of glucose in tissues (Salunkhe et al., 2016). In hypoxic environments, where oxygen availability is reduced, there is a shift toward a higher proportion of glucose metabolism (Goto et al., 2015), possibly affecting basal glucose and or glucose concentration following carbohydrate ingestion, and an increased proportion of glycolytic metabolism and fermentation can result in accumulating lactic acid production (Lottes et al., 2015).

A number of studies using reduced animal preparations suggest the possibility of altered glucose regulation in hypoxia, although with conflicting results. Using isolated islets of Langerhans beta cells from rats, Ota et al. (2012) found an attenuation of insulin secretion following 24 h of intermittent hypoxia, which may serve to maintain blood glucose in hypoxia in vivo. Conversely, using type II alveolar cells from rats, Ouiddir et al. (1999) found that GLUT1 transporters were upregulated following 18 h of hypoxic exposure, potentially facilitating an increase in glucose uptake in hypoxia. In addition, using dissected hindlimb muscles in rats, Cartee et al. (1991) showed an increase in glucose transporter translation following 40‐min perfusion with normocapnic anoxia (5% CO_2_ and 0% O_2_) perfusate.

Studies in humans investigating the specific effects of hypoxia on glucose concentration via interactions with insulin release or sensitivity and/or glucose utilization or storage remain limited and lack consensus. Many studies suggest that blood [glucose] may be increased in hypoxia, potentially through reductions in insulin secretion or sensitivity. Braun et al. (2001) found a reduction in insulin sensitivity in 12 women exposed to simulated 4,300 m for 16 h. Similarly, in 8 men brought immediately to 4,500 m for 7 days, Larsen et al. (1997) found an increase in both blood insulin and glucose in the first few days, suggesting the development of acute insulin insensitivity. Conversely, a number of studies suggest that blood [glucose] may be decreased in hypoxia, potentially through an increase in insulin secretion or sensitivity or increased glucose utilization. Kelly et al. (2010) found no difference in circulating insulin levels and/or a decrease in plasma glucose concentration in eight healthy humans exposed to hypobaric hypoxia equivalent to 4,300 m for 120 min. In 12 men exposed to a simulated altitude of 3,000 m, using tagged glucose and positron emission tomography, Chen et al. (2009) found an increase in plasma lactate levels and increased glucose uptake in cardiac muscle, but not skeletal muscle, brain or liver, suggesting the potential for differential mechanisms in glucose homeostasis in between tissues. In addition, Morishima and Goto (2014) found no differences in blood glucose or insulin concentrations in eight men exposed to an F_I_O_2_ of 14% over 7 h. These studies suggest that the mechanisms underlying the regulation of blood [glucose] may be counterbalanced, leaving [glucose] unchanged in hypoxia in humans (Morishima & Goto, 2014).

Given the low number of participants utilized in these limited number of previous studies in humans, in addition to the disparate findings and current lack of consensus regarding the mechanisms and effects of hypoxia on blood [glucose], we aimed to characterize the possible interaction between acute normobaric hypoxia and [glucose] following a standardized oral glucose beverage (75 g glucose in 296 ml) in a large number of healthy human participants. Specifically, we hypothesized that blood [glucose] over 80 min following acute glucose ingestion would not be significantly different between acute steady‐state normoxia and hypoxia conditions.

## 2 MATERIALS AND METHODS

### 2.1 Ethics and participant recruitment

Ethics approval was obtained in advance from the Mount Royal University Human Research Ethics Board (Protocol 2016‐91). This study abided by the Canadian Government Tri‐Council Policy on Research Ethics Policy Statement (TCPS2) and the Declaration of Helsinki, except for registration in the database. Participants were recruited for the experiment through word of mouth, and participants read and signed a consent form which included information on the study and risk information, a demographic form, which was used to record basic participant information (e.g., weight, height, and age) and confirms the lack of self‐reported medical conditions (e.g., respiratory, cardiovascular and metabolic). Participants had a body mass index (BMI) that fell within a healthy range of 20–25 kg/m^2^, were non‐smokers, and did not ingest any prescription medications, with the exception of oral contraceptives. No participants had been to altitude in the previous year and were residents of Calgary (1,130 m). Biological sex differences were not an a priori component of the study design in advance, and the ovarian cycle was not accounted for in our female participants.

### 2.2 Equipment and measurements

The Hypoxico Altitude Training System (Hypoxico, Inc.) complete with a small portable tent‐style chamber and nitrogen concentrator was used to simulate hypoxic conditions by displacing oxygen within the chamber, increasing the fraction of inspired nitrogen. Two participants at a time were seated inside the semi‐sealed chamber in chairs, with a small table between them for reading material/computers and use of portable devices (see below). A portable oxygen analyzer (MAXO_2_ME model R213P65, Maxtec) was used to detect and measure the fraction of inspired oxygen (F_I_O_2_) inside of the tent to ensure accuracy of the targeted level of oxygen during each trial. Chamber temperature was measured using a digital thermometer inside the chamber (VWR, Traceable Digital Thermometer), and recorded manually.

Blood [glucose] measurements were obtained using a standard capillary draw using sterile lancets and measured with an Accu‐Chek Aviva (Roche Diabetes Care) device with glucometer sensor strips. Trained investigators utilized capillary samples by asking participants to slip their hand out of the tent‐style hypoxic chamber (through an open zipper) for the ease of capillary sampling by a trained investigator positioned outside the chamber, for a total of nine separate samples per participant, per trial. After participant training, resting heart rate (HR) and peripheral blood oxygen saturation (SpO_2_) were measured by participants inside the chamber (PureSAT model 7500 pulse oximeter, Nonin Medical, Inc.). A single arterial blood pressure value was obtained using an automated brachial blood pressure cuff (Omron Healthcare), with mean arterial pressure calculated as 2/3 diastolic +1/3 systolic. In a subset of participants (*n* = 16), steady‐state pressure of end‐tidal CO_2_ (P_ET_CO_2_) was measured with a portable capnometer (Masimo, EMMA). For all measures at all time points, these measures represent a single measurement per participant. Participants were monitored through a window in the tent, and instructions were relayed to participants as required. For participant safety, participants were also provided with sheets with the Lake Louise acute mountain sickness (AMS) scoring system (Roach et al., 1993), and asked to report any acute AMS symptoms to investigators at each measurement time‐point.

### 2.3 Experimental protocol

Participants were recruited for a large, within‐individual cross‐over design study to compare the effects of normoxic and steady‐state hypobaric hypoxia on blood [glucose] over 80 min. On separate days, separated by at least 2 days, the control and experimental protocols were randomized for each participant. Participants visited the lab in a fasted state, having been instructed to avoid strenuous exercise (e.g., Knudsen et al., 2014) and caffeine and food for at least 12 h prior to beginning the experiment, which took place before noon on the testing day.

#### 2.3.1 Normoxic conditions

For the hypoxic trial, the nitrogen concentrator was running but not connected to the hypoxia tent. This arrangement provided a normoxic condition, with the participant breathing room air at an altitude of ~1,130 m (P_ATM_ O_2_ ≅ 140 mmHg, F_I_O_2_ = 0.209 of 669 mmHg in Calgary). Each participant was seated inside the semi‐sealed hypoxia chamber and underwent a 10‐min baseline measurement, where HR, SpO_2_, BP, P_ET_CO_2_, and blood [glucose] values were obtained. The participant was then allowed up to 5 min to ingest a standard glucose beverage (75 g, 296 ml, Trutol). After ingestion, the participant remained seated in the hypoxia tent for an additional 80 min, where glucose values were obtained every 10 min between 5 and 10, 15 and 20, 25 and 30, 35 and 40, 45 and 50, 55 and 60, 65 and 70, and 75 and 80‐min post‐glucose ingestion. F_I_O_2_ was monitored continuously every 5 min during the 80‐min period. SpO_2_, HR, MAP, and P_ET_CO_2_ values were obtained at 10, 20, 30, 40, 50, 60, 70, and 80‐min post‐glucose ingestion.

#### 2.3.2 Hypoxic conditions

The above protocol was replicated under hypoxic conditions. The nitrogen concentrator was used to achieve the hypoxic conditions in the tent, which was monitored and equivalent to an altitude of ~4,100 m (P_ATM_ O_2_ ≅ 100 mmHg, ~F_I_O_2_ = 0.148–0.15 of 669 mmHg in Calgary; ~1,130 m). SpO_2_ was monitored continuously for safety and did not fall below 80% in any participant in any trial.

### 2.4 Data and statistical analysis

The values obtained from both the control protocol (normoxia) and experimental protocol (hypoxia) are presented as mean values ± standard deviation (*SD*). Statistical significance was assumed at *p* < 0.05 Systat SigmaPlot (v. 14).

A paired *t* test was performed on mean values to compare data from the baseline period during both normoxia and hypoxia and trials for the following variables: F_I_O_2_, SpO_2_, HR, MAP, and fasted blood [glucose] (Table 1).

**TABLE 1:** Normoxia versus hypoxia intervention and participant baseline data recorded at the end of a 10‐min baseline in both normoxic and hypoxic conditions

Variable/Condition	Normoxia	Hypoxia
P_ATM_ (mmHg)	~669	~669
F_I_O_2_ (%)	20.4 ± 0.4	14.8 ± 0.6^a^
P_ATM_ O_2_ (mmHg)	~140	~100
P_I_O_2_ (mmHg)	~130	~92
SpO_2_ (%)	96.8 ± 2.2	88.6 ± 3.7^a^
HR (min^−1^)	72.0 ± 11.3	79.0 ± 8.9^a^
MAP (mmHg)	86.8 ± 8.0	88.2 ± 7.6
Fasted BG (mmol/L)	4.8 ± 0.4	4.9 ± 0.4

Note

P_ATM_, atmospheric pressure in the lab. F_I_O_2_, fraction of inspired O_2_ (%; measured). P_ATM_ O_2_, atmospheric O_2_ pressure (P_ATM_ × F_I_O_2_). P_I_O_2_, pressure of inspired O_2_ ((P_ATM_ – 47) × F_I_O_2_). SpO_2_, peripheral oxygen saturation (%; measured). HR, heart rate (min^−1^). MAP, mean arterial pressure (mmHg; measured). BG, blood glucose (mmol/L; measured). Values are reported as mean ± *SD*.

^a^ Denotes significant difference from normoxia for that variable (*p* < 0.001).

**FIGURE 1:**
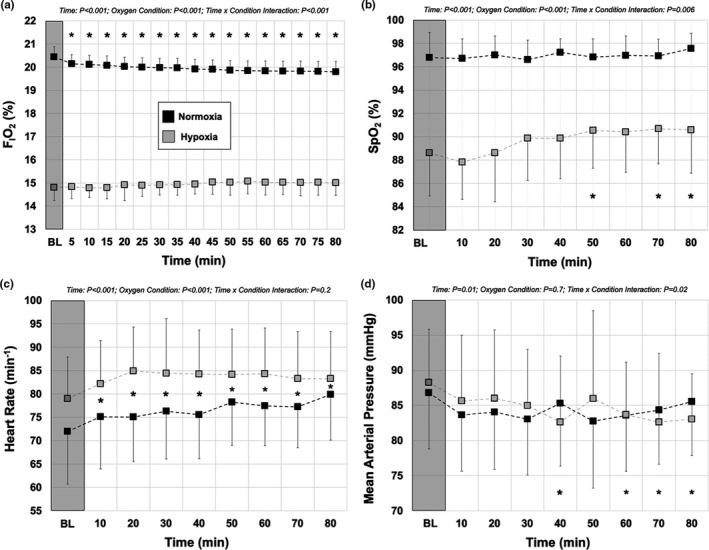
Changes in the fraction of inspired oxygen (F_I_O_2_; %), peripheral oxygen saturation (SpO_2_; %), heart rate (min^−1^), and mean arterial pressure (MAP; mmHg) in response to varying levels of oxygen over 80 min following glucose ingestion. The data from normoxic (black) and hypoxic (grey) conditions collected before (BL; transparent grey box) and after a glucose load. The baseline (BL) measures were obtained following 10 min in the chamber in each oxygen condition, but were fasted (transparent grey box). Participants then consumed the 75 g glucose beverage, under both oxygen conditions (see Figure 2). (a) Mean F_I_O_2_ values at baseline were lower in hypoxia (14.8 ± 0.6%) compared to normoxia (20.4 ± 0.5%; Table 1), and stable throughout the 80‐min protocol. (b) Mean SpO_2_ values at baseline were lower in hypoxia (88.6 ± 3.7%) compared to normoxia (96.8 ± 2.2%; Table 1). SpO_2_ values were stable throughout the 80‐min protocol in normoxia, but significantly higher than baseline during the hypoxia protocol. (c) Mean heart rate (HR) values at baseline were higher in hypoxia (79.0 ± 8.9 min^−1^) compared to normoxia (72.0 ± 11.3 min^−1^; Table 1). HR significantly increased from baseline in both normoxia and hypoxia trials. (d) Average MAP values at baseline were not different between hypoxia (88.2 ± 7.6 mmHg) and normoxia (86.8 ± 8.0 mmHg; Table 1). MAP values were relatively stable throughout the 80‐min protocol in normoxia and hypoxia, but were significantly reduced near the end of the hypoxia trial only. All *p* values for main effects (Time and Condition), and interaction (Time × Condition) indicated on each graph. *represents values different from baseline for that (or both) oxygen condition(s) over time. Values are reported as mean ± *SD*

Two factor repeated‐measures ANOVAs (factor one: time, factor two: oxygen condition) were performed to assess potential differences in F_I_O_2_, SpO_2_, HR, and MAP for each gas trial (i.e., normoxia and hypoxia) to assess potential changes in these variables over the protocol duration in each trial (Figure 1). Where significant F ratios were detected, a Student–Newman–Keuls post hoc test was utilized for pair‐wise comparisons.

**FIGURE 2:**
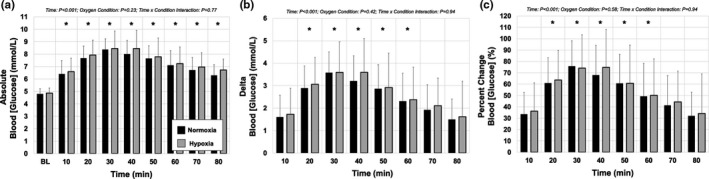
Blood [glucose] response at baseline and following a 75 g, 296 ml of standardized glucose beverage under both normoxic (black) and hypoxic (grey) conditions. (a) Absolute blood [glucose] values in response to glucose ingestion were not significantly different between normoxia and hypoxia. (b) Delta blood [glucose] values in response to glucose ingestion were not significantly different between normoxia and hypoxia. (c) Percent change blood [glucose] values in response to glucose ingestion were not significantly different between normoxia and hypoxia. All *p* values for main effects (Time and Condition), and interaction (Time × Condition) indicated on each graph. *represents values different from baseline for that (or both) oxygen condition(s) over time. Values are reported as mean ± *SD*

Two factor repeated‐measures ANOVAs (factor one: time, factor two: oxygen condition) were performed to assess potential differences in blood [glucose] between normoxic and hypoxic conditions. Where significant *F* ratios were detected, a Student–Newman–Keuls post hoc test was utilized for pair‐wise comparisons. These comparisons were carried out on absolute, delta from baseline, and percent change from baseline measures (Figure 2).

**FIGURE 3:**
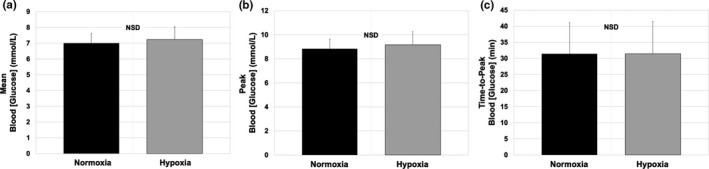
Average and peak blood [glucose], and time‐to‐peak [glucose] for all participants in both normoxic (black) and hypoxic (grey) conditions. (a) 80‐min average blood [glucose] was not significantly different between normoxia and hypoxic conditions (*p* = 0.21). (b) Absolute peak blood [glucose] was not significantly different between normoxia and hypoxic conditions (*p* = 0.14). (c) Time‐to‐peak [glucose] (from b) in minutes were not significantly different between normoxia and hypoxic conditions (*p* = 1.0). Values are reported as mean ± *SD*. NSD, no statistical difference

Paired *t* tests were utilized to compare the 80‐min average and absolute peak value of blood glucose (wherever it occurred) between hypoxia and normoxia. In addition, a paired *t* test was performed to compare the time‐to‐peak glucose concentration between hypoxia and normoxia (Figure 3).

To assess the potential relationship between SpO_2_ and absolute peak blood [glucose], we performed a Pearson product‐moment correlation between the peak glucose concentration in hypoxic conditions (wherever it occurred) and the corresponding SpO_2_ at that time point.

Last, to assess potential sex differences in [glucose] regulation in hypoxia, we utilized unpaired *t* tests to assess differences in mean, peak, and time‐to‐peak [glucose] in hypoxia between males and females.

## 3 RESULTS

### 3.1 Baseline values

Twenty‐eight participants (16 females; 21.8 ± 1.6 years; BMI 22.8 ± 2.5 kg/m^2^) completed the experiment in both randomized, within‐individual, cross‐over normoxic, and hypoxic conditions for a total of 56 trials. Baseline measurements for F_I_O_2_, SpO_2_, HR, MAP, and blood glucose were taken for each trial (Table 1). As expected, F_I_O_2_ and SpO_2_ were significantly lower in hypoxia compared to normoxia (*p* < 0.001), confirming the steady‐state hypoxic conditions. HR was significantly higher in hypoxia compared to normoxia (*p* < 0.001), confirming the hypoxic stimulus throughout. However, MAP and fasted blood [glucose] were not significantly different between hypoxic and normoxic trials (*p* = 0.33 and *p* = 0.47, respectively). Participants did not report any AMS symptoms in either trial.

### 3.2 Environmental measurements

F_I_O_2_ decreased mildly but significantly from 20.4 ± 4.5% to 19.8 ± 4.5% during the 80‐min normoxic trial (*p* < 0.001) but remained stable between 14.8 ± 0.6% and 15.0 ± 0.54% in the hypoxic trial (Figure 1a), confirming the hypoxic stimulus throughout. Under normoxic and hypoxic conditions, the chamber temperature was significantly higher at 80 min compared to baseline. Under normoxic conditions, the chamber temperature increased from 24.6 ± 1.5°C to 26.5 ± 1.5°C (*p *< 0.01), while under hypoxic conditions, the temperature increased from 25.4 ± 1.5°C to 27.0 ± 1.5°C (*p *< 0.01; data not shown).

### 3.3 Respiratory and cardiovascular measurements

Under normoxic conditions, P_ET_CO_2_ at 80 min was significantly higher (36.4 ± 8.9 mmHg) compared to baseline (31.8 ± 2.3 mmHg; *p *= 0.03), confirming a slight accumulation of chamber CO_2_. Under hypoxic conditions, P_ET_CO_2_ at 80 min was not significantly different at 30.8 ± 6.4 mmHg compared to normoxia 29.4 ± 4.0 mmHg (*p *= 0.29), suggesting the airflow from the nitrogen concentrator helped wash out metabolically derived CO_2_ in the chamber (P_ET_CO_2_ data not shown).

SpO_2_ was significantly higher in normoxic and hypoxic conditions throughout 80 min, confirming the hypoxic stimulus throughout (*p* < 0.001; Figure 1b). Although the SpO_2_ was stable across 80 min in the normoxic trial, it did increase significantly in the hypoxic condition by 70–80 min (interaction: *p *= 0.006). HR was higher in hypoxic than normoxic conditions throughout 80 min, suggesting a sympathetic response to hypoxia (*p *< 0.001; Figure 1c). However, HR increased significantly under both normoxic and hypoxic conditions over 80 min (*p *< 0.001). There were no differences in MAP between trials initially (*p *= 0.7; Figure 1d). However, MAP was stable during the normoxic trial, but decreased significantly over the duration of the hypoxic trial (*p *= 0.01).

### 3.4 Blood [glucose] measurements

Although blood [glucose] increased, peaked, and then decreased again over time following oral glucose consumption, there were no differences in absolute (interaction: *p *= 0.77; Figure 2a), delta from baseline (interaction: *p* = 0.94; Figure 2b) or percent changes in [glucose] (interaction: *p* = 0.94; Figure 2c) between oxygen conditions over the 80 min.

The 80‐min average blood [glucose] values for normoxia and hypoxia were 7.0 ± 0.6 and 7.3 ±0.8 mmol/L, respectively (*p* = 0.21; Figure 3a). The absolute peak values (wherever they occurred) for normoxia and hypoxia were 8.8 ± 0.8 and 9.2 ± 1.1 mmol/L, respectively (*p *= 0.14; Figure 3b). The time‐to‐peak blood [glucose] for normoxia and hypoxia were 31.4 ± 9.7 and 31.4 ± 10.1 min, respectively (*p* = 1.0; Figure 3c). Last, there was no correlation between SpO_2_ and peak blood [glucose] under hypoxic conditions (*r* = 0.013, *p *= 0.95; data not shown).

To address the possibility of sex differences in the [glucose] response in hypoxia, we compared the mean, peak, and time‐to‐peak between males (*n* = 12) and females (*n* = 16) during hypoxia. We found no differences in mean (*p* = 0.79), peak (*p* = 0.82) nor time‐to‐peak (*p* = 0.92) [glucose] values between males and females following glucose ingestion in hypoxia.

## 4 DISCUSSION

There are conflicting reports in the literature regarding the effects of hypoxia on mechanisms that may affect blood [glucose]. Accordingly, we sought to clarify the possible effect of acute normobaric hypoxia on glucose concentration in healthy humans with a larger sample size than previous reports. We hypothesized that blood [glucose] would not be significantly different between hypoxia and normoxia, as there are potential competing mechanisms affecting blood glucose regulation. The principal finding of this study is that the blood [glucose] response to an acute oral glucose load was not different between acute normobaric normoxic and hypoxic conditions in young healthy individuals over 80 min.

### 4.1 Hypoxia and blood [glucose]

Hypoxic conditions equivalent to altitudes greater than 4,000 m have been demonstrated to significantly lower blood [glucose] compared to normoxia during the first 60 min following 75 g oral glucose ingestion (*n* = 8; Kelly et al., 2010). Glucose uptake has also been shown to increase to match the metabolic need, decreasing blood [glucose] independent of [insulin] under hypoxic conditions Cartee et al., 1991; Chen et al., 2009; Kelly et al., 2010; Ouiddir et al., 1999). However, the main finding of this study, in a large cohort of healthy humans, suggests that blood [glucose] remains unchanged in acute steady‐state hypoxia when compared to normoxia. Conversely, hypoxia has also been shown by some studies to have inhibitory effects on insulin release and sensitivity (Braun et al., 2001; Larsen et al., 1997), which could serve to maintain or increase blood [glucose]. One possible mechanism leading to the inhibitory effects of hypoxia on both insulin sensitivity and secretion is the activation of the sympathetic nervous system. Stress hormones such as epinephrine have been shown to inhibit insulin‐induced glucose uptake, resulting in higher blood [glucose] (Chiasson et al., 1981). However, Braun et al. (2001) showed that the decrease in insulin sensitivity under hypoxic conditions was not epinephrine‐mediated. Cortisol, another stress hormone released upon exposure to hypoxia, is associated with a decrease in insulin secretion, possibly leading to higher blood [glucose] (Kamba et al., 2016). Thus, the overall lack of difference in blood [glucose] between conditions in our study may be explained in part due to the counterbalancing action of an increase in glucose uptake in non‐insulin‐dependent tissues (e.g., Watson & Pessin, 2001) or glucose utilization by somatic cells, and reduced insulin secretion and/or sensitivity, serving to maintain blood [glucose]. In addition, a study by Morishima and Goto (2014) showed (a) an increase in metabolic rate but (b) no significant differences in blood [glucose] over 7 h at a moderate simulated altitude (15% F_I_O_2_) following the ingestion of a 75 g oral glucose load, similar to the present study, although with a much lower number of participants (*n* = 8; Morishima & Goto, 2014).

### 4.2 Sympathetic response to acute hypoxia

The increase in HR observed over 80 min in the hypoxic trial is likely a response to the hypoxic stressor, although there was also an initial increase in HR in the normoxic trial. A possible explanation for these results is the activation of the sympathetic nervous system in response to the glucose ingestion itself (Smorschok et al., 2018; Synowski et al., 2013). Hypoxia is known to stimulate the peripheral chemoreceptors to elicit a sympathetic chemoreflex, which leads to an increase in HR and vascular resistance (i.e., vasoconstriction; Steinback et al., 2009). An increase in temperature has also been shown to increase sympathetic activation, leading to an increase in HR (Madaniyazi et al., 2016), as does glucose ingestion, which was recently shown to activate the sympathetic nervous system (Smorschok et al., 2018), leading to an increase in the heart rate (Synowski et al., 2013). However, MAP was significantly lower after 80 min in the hypoxic trial. This is likely due to hypoxia‐induced vasodilation (Blitzer et al., 1996). Accordingly, the vasodilatory effect of hypoxia is likely greater than its effects on sympathetic activation, lowering MAP over time.

### 4.3 Methodological considerations

The present study exposed participants to a normobaric hypoxic stimulus equivalent to a simulated altitude of ~4,100 m (P_ATM_ O_2_ ≅ 100 mmHg, ~F_I_O_2_ = 0.15 of 669 mmHg in Calgary; ~1,130 m) for only 80 min, shorter than a typical clinical oral glucose tolerance test (OGTT) protocol of 2 h. However, we do not believe that a longer duration would have had a significant effect on these responses, in part because participants were long past their peak blood [glucose] (occurring early at ~30 min), and Morishima and Goto (2014) demonstrated that there were no significant changes in glucose regulation after being exposed to hypoxia for 7 h. However, available evidence on glucose uptake by tissues is inconclusive regarding time course (e.g., Gamboa et al., 2011; Heinonen et al., 2011), and thus more studies are necessary in order for definite conclusions to be drawn regarding potential oxygen and blood [glucose] interactions. We aimed to assess the mean and peak blood [glucose] responses to acute hypoxia in healthy men and women, not assess the longitudinal responses in a clinical diabetic population, where the end‐point at 120‐min OGTT are utilized. Accordingly, we believe our protocol comparing normoxic and hypoxic peak and mean responses over 80 min in a large number of healthy participants addressed our experimental question on the effects of acute hypoxia on short‐term control of blood [glucose].

One limitation of this study was the lack of additional measurements of associated humoral variables. Lactate, insulin, and stress hormones (e.g., epinephrine and cortisol) were not measured, as we chose to trade off study complexity in favor of large participant recruitment. The measurement of plasma insulin may have confirmed the inhibitory effects of hypoxia on insulin secretion, and the measurements of stress hormones could suggest a mechanism resulting in an inhibitory effect on insulin secretion. Because of these limitations, we can only speculate as to the underlying mechanisms responsible for our observations, where blood [glucose] was unchanged between conditions. However, we believe that the high sample size and multiple methods of analysis (i.e., absolute, delta, percent, mean, peak, and time‐to‐peak) in our study add confidence to our conclusion that blood [glucose] homeostasis, and likely insulin release or sensitivity, is unchanged in healthy humans exposed to acute steady‐state normobaric hypoxia.

Our participants visited the lab fasted, having avoided strenuous exercise (Knudsen et al., 2014), caffeine, and food for at least 12‐h prior to visiting the lab on the morning of the testing day. However, we did not instruct participants regarding standardizing diet the night before. Robertson et al. (2002) demonstrated that the effects of high‐fat and high‐carbohydrate evening meals can persist overnight, affecting the blood [glucose] responses to an OGTT. However, they did not detect any effects on morning glucose or insulin concentrations. Although we acknowledge this weakness in our design, we did not observe any differences in blood [glucose] between groups under baseline conditions, and the large within‐participant likely minimizes any effects of high fat or high carbohydrate meals the day before our study.

The Accu‐Chek Aviva point‐of‐care device used in the present study utilizes the glucose‐1‐dehydrogenase method of measuring blood [glucose]. This method differs from similar studies that use laboratory determinations of blood [glucose] with the glucose oxidase method (Kelly et al., 2010; Morishima & Goto, 2014). However, given that our study was being conducted under hypoxic conditions, using a method other than the glucose oxidase method may be a strength of our study (see Tonyushkina & Nichols, 2009). Nonetheless, the Accu‐Chek Aviva has been assessed to be an accurate method of measuring blood [glucose] (Tack et al., 2012), and likely did not result in relevant inaccuracies. In addition, participants acted as their own controls, and we tracked changes over time, within‐individuals.

The small normobaric chamber used in the protocol also presents a limitation. The small decrease in F_I_O_2_ observed across the 80‐min normoxic trial is most likely due to the metabolic rate of the participants, utilizing the available ambient O_2_ and accumulating metabolically‐derived CO_2_ in the small chamber without air circulation. In addition, the small size of the normobaric chamber led to an accumulation of CO_2_ as well as an increase in temperature inside of the chamber. Both of these consequences likely contributed to the increases in sympathetic nervous system activation independent of the hypoxia, as evidenced by the increase in HR over 80 min. The accumulation of CO_2_ was assessed through the measurement of P_ET_CO_2_, which was mildly but significantly higher over 80 min compared to baseline in the normoxic trial. The accumulation of the CO_2_ in the chamber likely augmented the hypoxic ventilatory response (e.g., Teppema & Dahan, 2010), which could increase the relative SpO_2_ for the participants over 80 min. Moreover, in the hypoxic trial, the continuous airflow from the nitrogen concentrator likely eliminating some of the accumulating CO_2_, as it was replacing the chamber air. Therefore, the P_ET_CO_2_ at 80 min in the hypoxic trial was not significantly different compared to the baseline. The small increase in SpO_2_ observed in the hypoxic trial over 80 min was likely due to a hypoxic ventilatory response, in which the participants were responding to the lower amount of O_2_ in the normobaric hypoxia chamber (Teppema & Dahan, 2010). However, there was a small but a significant rise in SpO_2_ in the normoxic trial suggesting that the accumulating CO_2_, and possibly the increase in ambient temperature, increased ventilation in the normoxic trial (Tsuji et al., 2019). Last, the small increase in temperature could also result in peripheral vasodilation, which could contribute to the slight decrease of MAP over the 80 min in hypoxia. Thus, small normobaric hypoxia chambers come with a number of caveats that should be considered in experimental design and interpretation of results.

### 4.4 Potential significance

There is considerable controversy as to whether or not hypoxia elicits a change in the regulation of blood [glucose]. Therefore, the rationale of this study was to clarify the potential interactions between acute normobaric hypoxia and blood [glucose] in a large number of healthy, unacclimatized lowlanders. According to our results, the time course and magnitude of the changes in blood [glucose] in response to a standard oral glucose load in young healthy individuals are not different during acute exposure to steady‐state hypoxia. This information could be of interest for trekkers ascending to high altitude, as it may provide insightful information regarding nutritional requirements in chronic hypoxia, particularly with superimposed exercise, where metabolic demand is increased. Trekkers ascending and residing at high altitude may have to adjust their carbohydrate intake to meet their metabolic needs, but the extent of this requirement remains unknown.

There are also disparate findings with respect to diabetic populations at altitude. Previous studies have demonstrated that sustained hypoxia lowers blood [glucose] through increasing glucose utilization, which could be beneficial for people with diabetes (e.g., Kelly et al., 2010). A study performed by Duennwald et al. (2013) found that the exposure to intermittent hypoxia (IH) over 1 h resulted in a reduction in blood [glucose], suggesting a potential benefit of IH to diabetic patients. However, hypoxia may also decrease insulin sensitivity (Larsen et al., 1997) and possibly exacerbate the danger of uncontrolled diabetes. In addition, a previous study demonstrated that hypoxia increased blood [glucose] (Singh et al., 2014), possibly through decreasing insulin production or sensitivity, which may also exacerbate the negative effects of uncontrolled diabetes (i.e., hyperglycemia). In a study performed by Vera‐Cruz et al. (2015) using hyperbaric oxygen therapy (i.e., exposure to high PO_2_), fasted plasma [glucose] and glucose tolerance to an OGTT was improved in diabetic patients, suggesting an oxygen‐dependent effect on blood [glucose] regulation in patients with diabetes. Interestingly, with respect to high altitude natives, Okumiya et al. (2016) found that highland natives in Tibet living at altitudes above 3,500 m were more susceptible to the development of glucose intolerance and diabetes, but this result likely has a number of lifestyles (e.g., physical activity, diet) factors contributing to it. These disparate results depend upon the degree and mode of hypoxia, as well as the population of interest. Thus, there is a critical need for more research toward understanding the potential interaction between oxygen status and blood glucose regulation, particularly regarding the differential patterns (e.g., intermittent, acute, sustained) and magnitude of hypoxia, and the differential rates of diabetes in highlander versus low lander populations (e.g., Koufakis et al., 2019; Woolcott et al., 2015).

## 5 CONCLUSIONS

In conclusion, in a large sample of young healthy male and female participants, blood [glucose] in response to standard oral glucose ingestion (75 g, 296 ml) was unchanged during exposure to acute steady‐state normobaric hypoxia (F_I_O_2_ ~14.8%) compared to room air over 80 min, using multiple metrics including mean, peak, and time‐to‐peak responses.

## COMPETING INTERESTS

None declared.

## AUTHOR CONTRIBUTIONS

Jason Chan, protocol design, student group leadership, data collection, data analysis, draft manuscript writing, edited, and approved the final manuscript; Alexandra Chiew, protocol design, data collection, data analysis, edited, and approved the final manuscript; Alexander Rimke, protocol design, data collection, data analysis, edited, and approved the final manuscript; Garrick Chan, protocol design, data collection, data analysis, edited, and approved the final manuscript; Zahrah Rampuri, data collection, edited, and approved the final manuscript; Mackenzie Kozak, data collection, edited, and approved the final manuscript; Normand Boulé, intellectual contribution, edited, and approved the final manuscript; Craig Steinback intellectual contribution, edited, and approved the final manuscript; Margie Davenport, intellectual contribution, edited, and approved the final manuscript; Trevor Day, ethics, funding, lab equipment support, intellectual contributions, protocol design, data analysis, edited, and approved the final manuscript; The corresponding author (TAD) confirms that all coauthors have reviewed and approved of the manuscript prior to submission.
